# Influence of Print Orientation on Surface Roughness in Fused Deposition Modeling (FDM) Processes

**DOI:** 10.3390/ma12233834

**Published:** 2019-11-21

**Authors:** Irene Buj-Corral, Alejandro Domínguez-Fernández, Ramón Durán-Llucià

**Affiliations:** School of Engineering of Barcelona (ETSEIB), Department of Mechanical Engineering, Universitat Politècnica de Catalunya (UPC), Avinguda Diagonal, 647, 08028 Barcelona, Spain; alejandro.dominguez-fernandez@upc.edu (A.D.-F.); ramon.duran@estudiant.upc.edu (R.D.-L.)

**Keywords:** Fused Deposition Modeling, roughness, Polylactic Acid, print orientation angle, build angle

## Abstract

In the present paper, we address the influence of print orientation angle on surface roughness obtained in lateral walls in fused deposition modelling (FDM) processes. A geometrical model is defined that considers the shape of the filaments after deposition, in order to define a theoretical roughness profile, for a certain print orientation angle. Different angles were considered between 5° and 85°. Simulated arithmetical mean height of the roughness profile, Ra values, were calculated from the simulated profiles. The Ra simulated results were compared to the experimental results, which were carried out with cylindrical PLA (polylactic acid) samples. The simulated Ra values were similar to the experimental values, except for high angles above 80°, where experimental roughness decreased while simulated roughness was still high. Low print orientation angles show regular profiles with rounded peaks and sharp values. At a print orientation angle of 85°, the shape of the profile changes with respect to lower angles, showing a gap between adjacent peaks. At 90°, both simulated and experimental roughness values would be close to zero, because the measurement direction is parallel to the layer orientation. Other roughness parameters were also measured: maximum height of profile, Rz, kurtosis, Rku, skewness, Rsk, and mean width of the profile elements, Rsm. At high print orientation angles, Rz decreases, Rku shifts to positive, Rsk slightly increases, and Rsk decreases, showing the change in the shape of the roughness profiles.

## 1. Introduction

In the fused deposition modelling (FDM) process, a filament is heated and then the material is deposited by a nozzle onto a printing bed. FDM printed parts are used in different applications, for example medical, electrical, aerospace, etc. For example, it allows printing patterns for investment casting of biomedical implants [1]. In addition, highly metallic-filled conductive composites can be prepared by FDM to be used in electromagnetic shielding, sensors, and circuit printing [2]. As for aerospace, carbon fiber reinforced PLA printed composites can be used [3].

FDM allows a wide range of materials, and the printed parts have effective mechanical properties. However, printing speed is low and the layer-by-layer building of parts leads to poor surface roughness due to the stair stepping effect [4,5]. When the lateral walls of a certain workpiece are inclined, the use of printing supports is required. In addition, the inclination of the lateral walls will have an effect on surface roughness, since the wall will not be perpendicular to the layer plane.

Different authors have studied the effects of printing parameters on surface roughness. For example, Pérez et al. [6] considered layer height, printing speed, temperature, printing path, and wall thickness. They found that layer height and wall thickness had the greatest influence on arithmetical mean height, Ra. Reddy et al. [7] used layer thickness, material infill, and printing quality as factors. They also considered build inclination. Both layer thickness and build inclination turned out to be the most influential factors on roughness. Peng and Yan [8] optimized roughness and energy consumption. They employed layer thickness, printing speed, and infill ratio as factors, with layer height being the most important parameter influencing roughness. Kovan et al. [9] studied the effect of layer height and printing temperature on surface roughness. You [10] studied infill ratio, printing temperature, and printing speed. They found that roughness increases with printing speed and decreases with infill ratio. Altan et al. [11] studied the effect of printing processes on surface roughness and tensile strength, with layer thickness and deposition head velocity being the most influential parameters on roughness. Mohamed et al. [12] investigated the effect of printing parameters on the dynamic mechanical properties of polycarbonate–acrylonitrile butadiene styrene (PC-ABS) printed parts. The main factors were layer height, air gap, and the number of contours. Luis studied Ra and Rq values obtained through experimental tests in FDM processes [13].

Regarding previous geometrical models for roughness in FDM processes, Pandey et al. obtained a semiempirical model for roughness, in which they took into account both layer thickness and build orientation [14]. Ahn et al. considered the filaments to have the shape of elliptical curves which overlap in the vertical direction [15]. Boschetto et al. approximated the roughness profiles of printed parts as a sequence of circumference arcs [16]. Ding et al. obtained roughness profiles from the overlapping of different surfaces representing beads [17]. Kaji and Barari obtained roughness profiles from the cusp geometry of the lateral walls of parts, taking into account both straight lines and degree two polynomial curves [18]. On the other hand, the Slic3r manual considers the shape of the cross section of the deposited filaments to be a rectangle with round ends, in which the initial area of the filament is equal to its final area [19]. A similar approach was employed by Jin et al. However, the length of the rectangle in their cross-section model is calculated based on the volume conservation, taking into account the plastic flow-rate and speed-rate [20]. From the assumptions made in [19], Buj et al. calculated pore size from the nozzle diameter, infill, and layer height of printed samples [21]. Other authors take into account the overlapping among filaments, due to diffusion when printing high melting temperature thermoplastic polymers such as polyether ether ketone (PEEK) [22]. However, this effect is not so important with low melting temperature polymers like polylactic acid (PLA) and acrylonitrile butadiene styrene (ABS).

Regarding print orientation, Bottini and Boschetto investigated the effect of deposition angle and interference grade on the assembly and disassembly forces in the interference fit of FDM printed parts [23]. They found that assembly forces depend on both parameters, while disassembly forces do not depend on deposition angle, as surface morphology is modified as a result of assembly. In addition, different authors have studied the influence of print orientation on the mechanical strength of parts [24]. Domingo-Espín et al. studied six different orientations and determined stiffness and tensile strength of polycarbonate (PC) samples [25]. They recommended that, when the yield strength of a material is exceeded, the parts should be oriented in a way that the greater tensile stresses are aligned with the direction of the longest contours, in order increase their tensile strength. Knoop et al. studied the effect of building orientation on the tensile, flexural, and compressive strength of polyamide (PA) parts [26]. As a general trend, they found higher tensile strength for build orientation X of the tensile test specimens (on its edge), than for build orientation Y (flat lying), or Z (upright). Uddin et al. studied the effect of print orientation on the tensile and compressive strength of ABS parts [27]. They obtained the highest stiffness and failure strength for layer thickness 0.09 mm, printing plane YZ and horizontal print orientation. Chacón et al. [28] studied the influence of print orientation on the tensile and flexural strength of PLA parts. They observed that low layer thickness and high feed rate values improved mechanical performance. Sood et al. [29] investigated the effect of layer thickness, build orientation, raster angle, raster width, and air gap on the compressive strength of parts. They found that an artificial neural network (ANN) model was better for modeling compressive strength than a regression model. The optimal value for layer orientation, giving higher compressive strength, was 0.036°. McLouth et al. [30] analyzed the influence of print orientation and raster pattern on the fracture toughness of ABS parts. They concluded that samples with layers that are parallel to the crack plane turned out to have lower fracture toughness than samples with other print orientations. As for the influence of print orientation on roughness, Chaudhari et al. studied the surface finish of ABS parts printed with different layer thickness, infill, orientation, and postprocessing operation. They found that infill and postprocessing had the greatest influence on roughness [31]. Thrimurthulu et al. [32] simultaneously optimized surface roughness and build time, as a function of slice thickness and build deposition orientation. Both parameters influenced roughness. Wang et al. [33] studied the effects of: layer thickness, deposition style, support style, deposition orientation in the Z direction (build angle), deposition orientation in the X direction (raster angle), and build location on the tensile strength, dimensional accuracy, and surface roughness of printed parts. They observed that layer thickness was the most influential parameter.

The aim of the present paper is to define a geometrical model for surface roughness in lateral walls, in FDM printing processes. The model considers the different print orientations, with simulated results being compared to experimental results. To do so, cylindrical samples are printed with different print orientations of between 0° and 85°, in PLA. Roughness is measured along the generatrix of the samples, by means of a contact roughness meter. Then, the results from the model are compared to the experimental results for different print orientation angles.

## 2. Materials and Methods

### 2.1. Geometrical Model

**Figure 1:**
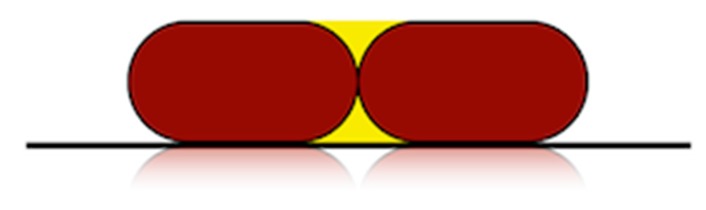
Schematic of the cross-section of two adjacent deposited filaments with print orientation angle of 0° (the horizontal line corresponds to the printing bed).

A geometrical model was defined to calculate roughness in lateral walls, for parts with different print orientations. Two assumptions were made (Figure 1):

- The shape of the cross-section of the filaments after deposition is a rectangle with rounded edges, with a semicircle at each side [19].
- There is no overlapping of adjacent filaments due to material diffusion, since processing temperatures are not excessively high.

**Figure 2:**
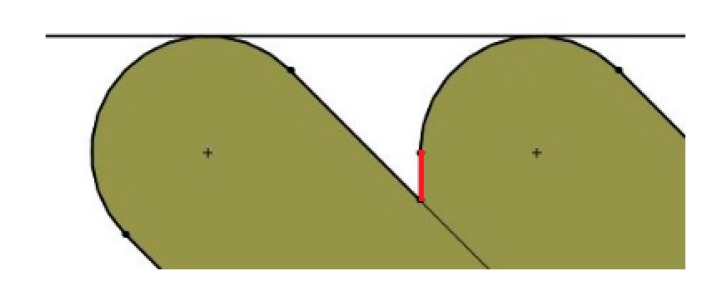
Schematic of the cross-section of two deposited filaments with print orientation angle of 45°.

**Figure 3:**
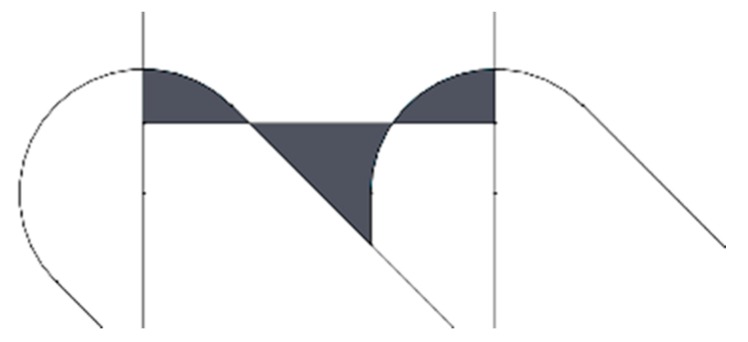
Profile for print orientation angle of 45°, with the areas highlighted in grey.

Considering these assumptions, arithmetical mean height Ra values were calculated for each print orientation, according to the following procedure:

1. The geometry of two deposited filaments (one on top of the other) is drawn for each print orientation studied, using the Solid Woks 2017 software (Dassault Systèmes Solidworks Corporation, Waltham, MA, USA). The tangent line at the edge of the two filaments is determined and the figure is rotated until the tangent line becomes a horizontal line. Figure 2 shows an example for print orientation angle of 45°.
2. The shape of the edges of the two filaments is considered to be the theoretical roughness profile of the lateral wall of the parts. In order to avoid profiles with negative draft angle from the vertical direction (which are not found in experimental roughness profiles), vertical lines are drawn in the area where the end of one filament adjoins the other filament, if necessary (see red line in Figure 2).
3. The total measurement length of the profiles was defined as the distance between the centers of the circumferences of the edges of the two layers (Figure 3).
4. The center line of the profiles was found with Solid Works, taking into account the mean value theorem for integrals. The center line divides a profile function into two parts, so that the areas contained by the profile above and below the center line are equal (Figure 3). The first mean value theorem for integrals says that for all continuous functions in the area [a, b] a point c exists within the interval [a, b], which makes the area below the function equal to its image at point c for all the interval length, according to Equation (1).
  (1) $$\left(b-a\right)\cdot f\left(c\right)=\int_{a}^{b}f\left(x\right)\;dx.$$
5. The arithmetical mean height roughness parameter Ra (in µm) was calculated according to Equation (2).
  (2) $$Ra=\frac{1}{L}\int_{0}^{L}\left|f\left(x\right)\right|\;dx$$
  where L is the measurement length in mm, and *f*(*x*) is the discrete function that defines the roughness profile, in mm.

In order to compare the simulated results of the model with the experimental results obtained with a contact roughness meter, the geometry of the roughness meter tip was added to the ideal geometry of the layers. Its cross-section was assumed to be an isosceles rectangle triangle of 1 mm height, with sharp edges.

**Figure 4:**
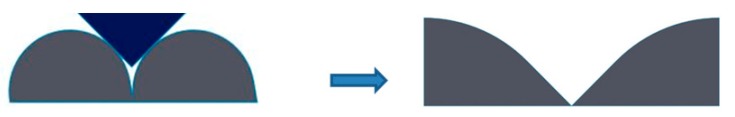
Schematic of the printed layers with the roughness tip, for print orientation angles higher than 45°.

**Figure 5:**
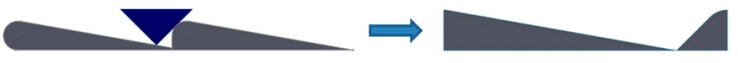
Representation of the roughness tip with the printed layers, for print orientation angle higher than 45°.

Two different cases were found:

(a) For print orientation angles lower or equal to 45°, the tip leans on two surfaces, and a new profile is obtained which shows shallower valleys than the previous one (Figure 4).
(b) For print orientation angles higher than 45°, the tip leans on one of the two sides of the profile. Moreover, it is not able to reach the lowest part of the profile (Figure 5). The modified valleys have the same depth as the original ones, but the shape of the profile changes.

New simulated Ra values were calculated from the modified profiles.

### 2.2. Printing Process

A double extruder Sigma printer from BCN3D Technologies (Barcelona, Spain) was used. Cylindrical PLA samples were printed, of 12.7 mm diameter and 25.4 mm height, according to a height-to-width ratio of 2.

Printing parameters are provided in Table 1 (Appendix).

Layer height is the thickness of each deposited layer. Infill ratio is the amount of solid material within the volume of a printed structure. Infill type was rectangular in all cases, with raster angle 0°. Air gap is the space between filaments, and depends on the infill ratio used. Shells are the layers that are printed around the infill area. No shell was printed in this case.

**Figure 6:**
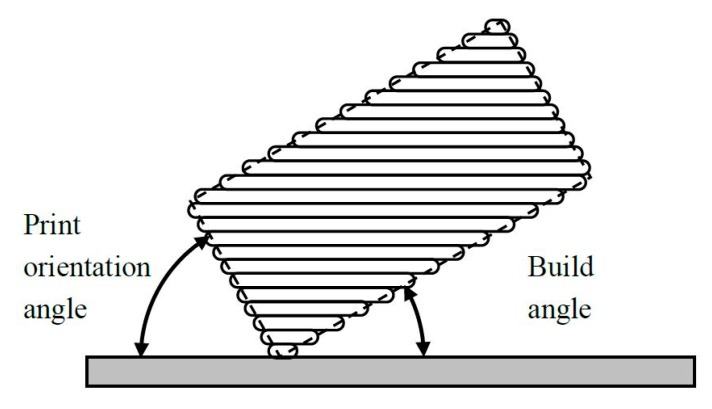
Schematic of a printed part with the print orientation angle and the build angle.

Print orientation angle and build angle are complimentary angles. They are shown in Figure 6.

### 2.3. Roughness Measurement

Roughness was measured in a contact Taylor Hobson Talysurf 2 roughness meter (AMETEK Inc., Berwyn, PA, USA), with two different Gaussian filters of cut-off 8 mm and 2.5 mm respectively. Several roughness parameters were taken into account: arithmetical mean height, Ra, maximum height of the profile, Rz, kurtosis, Rku, skewness, Rsk, and mean width of the profile elements, Rsm.

**Figure 7:**
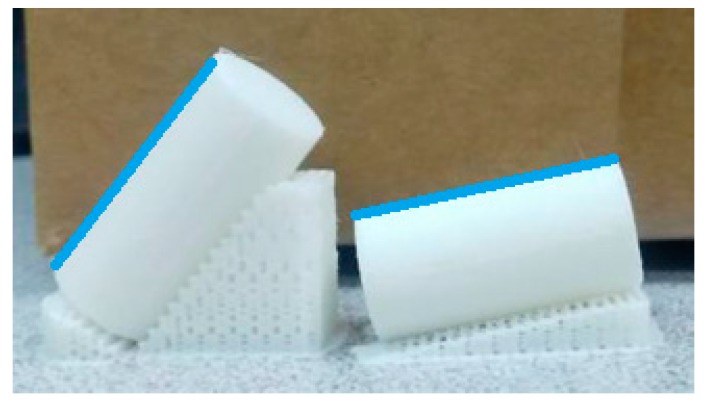
Printed specimens with the measurement direction highlighted in blue.

Measurement direction coincides with one generatrix of the cylinders, specifically the one that is placed opposite the printing supports. As an example, the blue lines in Figure 7 show the measuring direction of two specimens with different print orientation angles.

If a print orientation angle of 0° were considered, there would be no need to use printing supports. Thus, roughness would be measured along any generatrix of the specimen.

## 3. Results

### 3.1. Roughness Profiles

**Figure 8:**
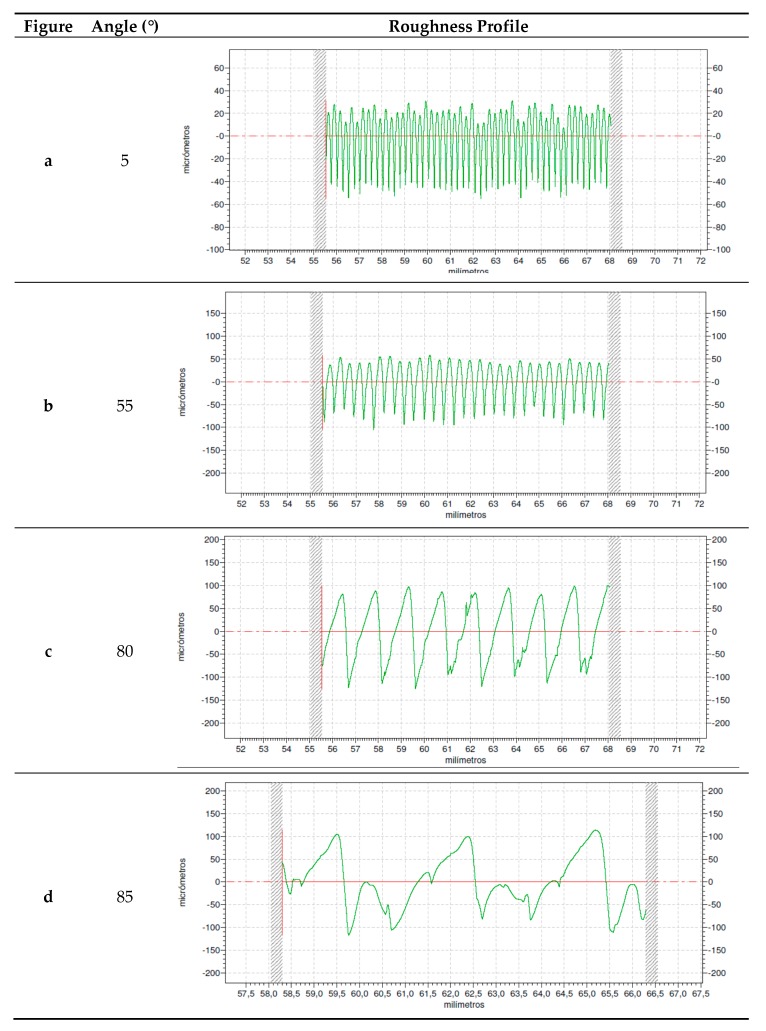
Roughness profiles for print orientation angle of: (**a**) 5°, (**b**) 55°, (**c**) 80°, and (**d**) 85°.

As an example, Figure 8 presents experimental roughness profiles for different print orientation angles. A print orientation angle of 5° (Figure 8a) corresponds to a regular profile, with the typical shape obtained in lateral walls when layers have no inclination, in FDM processes. The profile shows rounded peaks and sharp valleys, and the peak width corresponds to the layer height employed. As the angle increases, similar profiles are obtained, for example for a print orientation angle of 55° (Figure 8b). For a print orientation angle of 80°, a sawtooth shape is observed for the profile. For a print orientation angle of 85°, the profile becomes more irregular, combining high peaks for the filament edges with a transition flat area between consecutive peaks. The distance between peaks increases. At a print orientation angle of 90°, the layers would be parallel to the direction in which roughness is measured. For this reason, the theoretical roughness value would be zero.

**Figure 9:**
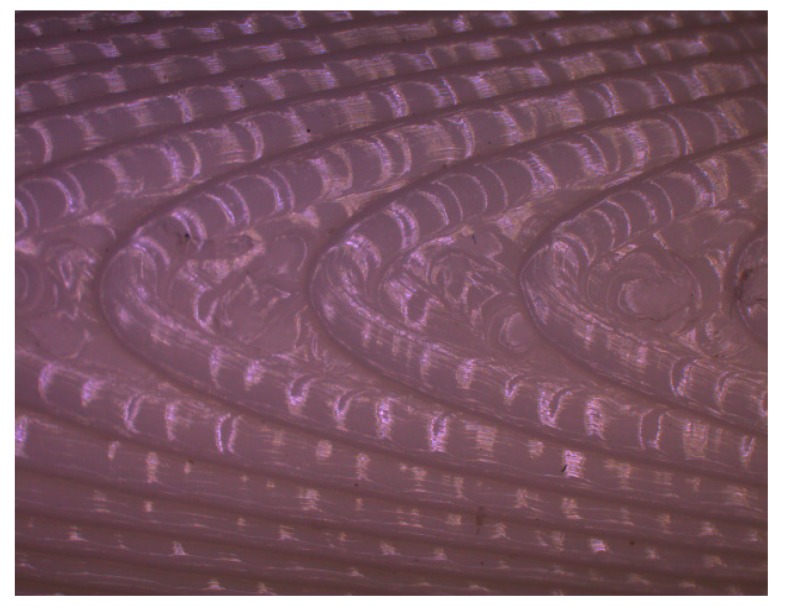
Plan view of a sample with print orientation angle of 85°.

Figure 9 shows a picture (plan view) of a sample manufactured with print angle of 85°.

As print orientation angle increases, the stair-stepping effect becomes more evident. It can be observed that the high inclination of layers leads to a greater distance between crests, with wide plateaus that provide lower roughness values. In addition, the measured roughness profile in this case is more irregular than the rest of the profiles (Figure 8d), causing greater discrepancy between experimental and simulated roughness values.

### 3.2. Roughness Values

**Figure 10:**
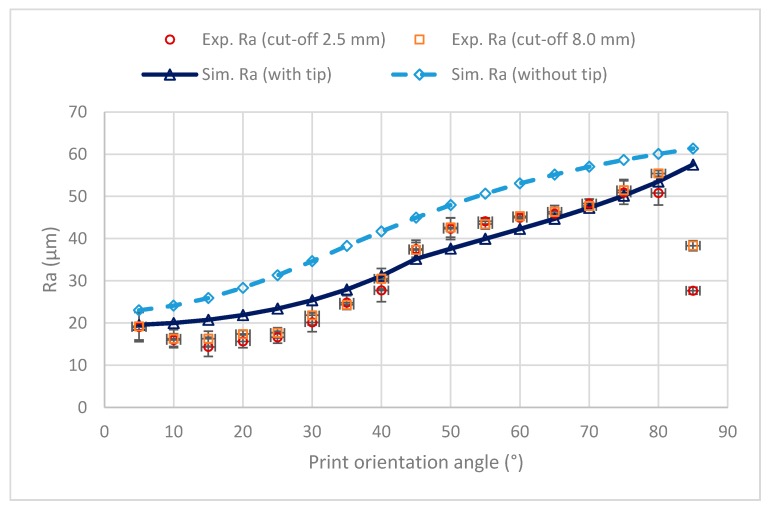
Arithmetical mean height (Ra) vs. print orientation angle.

Figure 10 presents the simulated Ra results, considering the tip geometry or not, as well as the measured roughness with cut-off of either 8 mm or 2.5 mm. According to ISO 4288 standard [31], a cut-off value of 2.5 mm is recommended for Ra values between more than 2 μm and 10 μm, and a cut-off value of 8 mm is recommended for Ra values higher than 10 μm. Error bars correspond to ± standard deviation values.

In all cases, as expected, the roughness results simulated with the tip were lower than those simulated without the tip, since the tip reduces the valley depth of the profile. As a general trend, the experimental values agree with the simulated values with tip up to a print orientation angle of 80°. The results agree with those of Reddy et al. [4], who found that Ra decreases with build angle, which is the complimentary angle of the print orientation angle. They found maximum Ra values of 50 µm for build angles of 10° (printing angle of 80°). However, in the present work, at 85° the experimental roughness decreases significantly with respect to 80°. Such decrease is more important for the cut-off of 2.5 mm than for the cut-off of 8 mm. This suggests that the abrupt transition from high simulated roughness values at the print orientation angle of 80° to the zero simulated roughness value at the print orientation angle of 90° is more gradual in the experimental tests.

In order to analyse the shape of the roughness profiles at high print orientation values, Table 2 provides the experimental values of other roughness parameters, Rz, Rsk, Rku, and Rsm, measured with a cut-off of 8 mm.

Rz increases with print orientation angle, as expected, up to 80°, and then decreases at print orientation angle of 85°. Skewness shows negative values up to 70°, corresponding to higher valleys than peaks (Figure 8b). At 75° and 80° skewness values are close to zero, corresponding to symmetric profiles (Figure 8c). At 85°, skewness has a positive value, with higher peaks than valleys (Figure 8d). Kurtosis is lower than 3 in all cases, pointing out that the peaks are sharper than those corresponding to a normal distribution of heights. At print orientation angle of 85°, the highest Rku value is obtained of 2.312, corresponding to rounder peaks and valleys. Parameter RSm, mean width of the profile elements, increases with print orientation angle, since the effective distance between layers increases. However, at 85° the parameter decreases, because small roughness peaks are measured in the gaps between adjacent peaks (Figure 8d).

## 4. Discussion

The proposed model allows simulating Ra values to be obtained in lateral walls of FDM printed parts. Unlike other models, which take into account overlapping among adjacent deposited filaments [15,17], the present model makes the assumption that printing temperature is low enough to avoid overlapping. It also assumes that the shape of the cross-section of the deposited filament is rectangular with rounded edges [19,20].

Experimental Ra values are similar to simulated ones at low print orientation angles, and they increase with print orientation angle as reported by Reddy et al. [7]. However, at high angles above 80°, the experimental roughness values are lower than the simulated ones. This suggests a gradual decrease in the experimental roughness between 80° and 90°. At 90°, the printing direction would be parallel to the measuring direction and, for this reason, the experimental roughness values would be close to zero.

At low print orientation angles, regular profiles are obtained with round peaks and sharp valleys, which are typical of FDM processes [34]. At a high print orientation angle of 85°, the distance between consecutive peaks increases, leading to a flat area or gap. In this case, not only the arithmetical mean height of the profile Ra decreases but the maximum height of profile Rz and the mean width of the profile Rsm. Skewness parameter Rsk becomes positive and kurtosis parameter Rku increases, noting the change in the profile shape [35].

In the future, a similar methodology using the mean value theorem for integrals, can will be applied to calculate simulated Ra in other manufacturing processes, either additive manufacturing processes or subtractive processes, provided that the theoretical geometry of the roughness profile can be obtained.

## 5. Conclusions

This paper presents a geometrical model for the simulation of roughness profiles obtained with different print orientation angles in FDM processes, in order to determine the mean height of the roughness profile, Ra. In addition, experimental tests were performed. The main conclusions of the paper are as follows:

- Use of the mean value theorem for integrals allows calculating Ra from the geometrical model of the roughness profile in a simple way. This methodology is also valid in case the assumptions of the model need to be varied, or even for other manufacturing processes.
- At low print orientation angles, regular profiles are obtained, in which peak amplitude corresponds to layer height. At high print orientation angles, peak width increases, with a flat area or gap between consecutive peaks.
- As a general trend, both simulated and experimental amplitude roughness values increase with print orientation angle, as the stair-stepping effect is accentuated. However, simulated roughness results decrease abruptly (simulated roughness would be zero at 90° because the roughness measurement direction coincides with the direction of the printed layers), while experimental results show a more gradual decrease starting at around 85°.
- At a high print orientation angle of 85°, skewness parameter Sku becomes positive, kurtosis parameter Rku increases, and the mean width of the profile Rsm shows a slight decrease with respect to 80°, thus noticing the change in the shape of the roughness profile.

## Figures and Tables

**Table 1:** Printing parameters of the experimental tests.

Parameter	Values
Layer height (mm)	0.25
Infill ratio (%)	50
Nozzle diameter (mm)	0.4
Printing speed (mm/s)	60
Printing temperature (°C)	205
Print orientation angle (°)	From 5 to 85

**Table 2:** Rz, Rsk, Rku, and Rsm values.

Print Angle (°)	Mean Value Rz (μm)	Standard Deviation Rz (μm)	Mean Value Rsk	Standard Deviation Rsk	Mean Value Rku	Standard Deviation Rku	Mean Value Rsm (μm)	Standard Deviation Rsm (μm)
50	161.211	17.050	−0.433	0.091	2.032	0.092	388.588	2.344
55	169.910	19.409	−0.372	0.039	1.875	0.097	437.863	1.618
60	187.277	7.030	−0.201	0.033	1.742	0.040	501.911	1.214
65	183.679	12.237	−0.121	0.050	1.748	0.033	593.597	2.417
70	196.639	3.787	−0.258	0.012	1.872	0.002	728.450	3.314
75	225.699	20.530	−0.153	0.103	1.788	0.058	963.619	3.645
80	237.129	10.838	0.035	0.104	1.791	0.017	1428.000	4.048
85	211.161	27.924	0.050	0.137	2.312	0.034	1226.810	8.924

## Appendix A

List of printing parameters

[profile]

layer_height = 0.25

wall_thickness = 1.2

retraction_enable = True

solid_layer_thickness = 1.2

fill_density = 50

print_speed = 60

print_temperature = 205

print_temperature2 = 205

print_temperature3 = 0

print_temperature4 = 0

print_temperature5 = 0

print_bed_temperature = 65

support = Everywhere

platform_adhesion = Raft

support_dual_extrusion = First extruder

wipe_tower = False

wipe_tower_volume = 50

ooze_shield = False

filament_diameter = 2.85

filament_diameter2 = 2.85

filament_diameter3 = 0

filament_diameter4 = 0

filament_diameter5 = 0

filament_flow = 100

nozzle_size = 0.4

retraction_speed = 40

retraction_amount = 6.8

retraction_dual_amount = 3

retraction_min_travel = 1.5

retraction_combing = No Skin

retraction_minimal_extrusion = 0

retraction_hop = 0.08

bottom_thickness = 0.2

layer0_width_factor = 100

object_sink = 0

overlap_dual = 0.15

travel_speed = 200

bottom_layer_speed = 35

infill_speed = 35

solidarea_speed = 35

inset0_speed = 35

insetx_speed = 35

cool_min_layer_time = 5

fan_enabled = True

skirt_line_count = 2

skirt_gap = 2

skirt_minimal_length = 150.0

fan_full_height = 0.5

fan_speed = 85

fan_speed_max = 100

cool_min_feedrate = 10

cool_head_lift = False

solid_top = True

solid_bottom = True

fill_overlap = 15

perimeter_before_infill = True

support_type = Lines

support_angle = 20

support_fill_rate = 50

support_xy_distance = 0.6

support_z_distance = 0.15

spiralize = False

simple_mode = False

brim_line_count = 5

raft_margin = 3.0

raft_line_spacing = 3.0

raft_base_thickness = 0.3

raft_base_linewidth = 1.0

raft_interface_thickness = 0.28

raft_interface_linewidth = 0.6

raft_airgap_all = 0.0

raft_airgap = 0.22

raft_surface_layers = 2

raft_surface_thickness = 0.15

raft_surface_linewidth = 0.4

fix_horrible_union_all_type_a = True

fix_horrible_union_all_type_b = False

fix_horrible_use_open_bits = False

fix_horrible_extensive_stitching = False

plugin_config = (lp1

  (dp2

  S’params′

  p3

  (dp4

  sS′filename′

  p5

  S’RingingRemover.py′

  p6

  sa.

object_center_x = −1

object_center_y = −1

## References

[1] Singh D., Singh R., Boparai K.S. (2018). Development and surface improvement of FDM pattern based investment casting of biomedical implants: A state of art review. J. Manuf. Process..

[2] Kwok S.W., Goh K.H.H., Tan Z.D., Tan S.T.M., Tjiu W.W., Soh J.Y., Ng Z.J.G., Chan Y.Z., Hui H.K., Goh K.E.J. (2017). Electrically conductive filament for 3D-printed circuits and sensors. Appl. Mater. Today.

[3] Tian X., Liu T., Yang C., Wang Q., Li D. (2016). Interface and performance of 3D printed continuous carbon fiber reinforced PLA composites. Compos. Part A Appl. Sci. Manuf..

[4] Gibson I., Rosen D., Stucker B. (2014). Additive Manufacturing Technologies: 3D Printing, Rapid Prototyping, and Direct Digital Manufacturing.

[5] Ngo T.D., Kashani A., Imbalzano G., Nguyen K.T.Q., Hui D. (2018). Additive manufacturing (3D printing): A review of materials, methods, applications and challenges. Compos. Part B Eng..

[6] Pérez M., Medina-Sánchez G., García-Collado A., Gupta M., Carou D. (2018). Surface quality enhancement of fused deposition modeling (FDM) printed samples based on the selection of critical printing parameters. Materials.

[7] Reddy V., Flys O., Chaparala A., Berrimi C.E., V A., Rosen B. (2018). Study on surface texture of Fused Deposition Modeling. Procedia Manuf..

[8] Peng T., Yan F. Dual-objective Analysis for Desktop FDM Printers: Energy Consumption and Surface Roughness. Proceedings of the Procedia CIRP.

[9] Kovan V., Tezel T., Topal E.S., Camurlu H.E. (2018). Printing Parameters Effect on Surface Characteristics of 3D Printed Pla Materials. Mach. Technol. Mater..

[10] You D.H. (2016). Optimal printing conditions of PLA printing material for 3D printer. Trans. Korean Inst. Electr. Eng..

[11] Altan M., Eryildiz M., Gumus B., Kahraman Y. (2018). Effects of process parameters on the quality of PLA products fabricated by fused deposition modeling (FDM): Surface roughness and tensile strength. Mater. Test..

[12] Mohamed O.A., Masood S.H., Bhowmik J.L. (2017). Characterization and dynamic mechanical analysis of PC-ABS material processed by fused deposition modelling: An investigation through I-optimal response surface methodology. Meas. J. Int. Meas. Confed..

[13] Luis Pérez C.J. (2002). Analysis of the surface roughness and dimensional accuracy capability of fused deposition modelling processes. Int. J. Prod. Res..

[14] Pandey P.M., Reddy N.V., Dhande S.G. (2003). Improvement of surface finish by staircase machining in fused deposition modeling. J. Mater. Process. Technol..

[15] Ahn D., Kweon J.H., Kwon S., Song J., Lee S. (2009). Representation of surface roughness in fused deposition modeling. J. Mater. Process. Technol..

[16] Boschetto A., Giordano V., Veniali F. (2012). Modelling micro geometrical profiles in fused deposition process. Int. J. Adv. Manuf. Technol..

[17] Ding D., Pan Z., Cuiuri D., Li H., Van Duin S., Larkin N. (2016). Bead modelling and implementation of adaptive MAT path in wire and arc additive manufacturing. Robot. Comput. Integr. Manuf..

[18] Kaji F., Barari A. (2015). Evaluation of the Surface Roughness of Additive Manufacturing Parts Based on the Modelling of Cusp Geometry. IFAC-PapersOnLine.

[19] Hodgson G., Ranelluci A., Moe J. (2016). Slic3r Manual—Flow Math.

[20] Jin Y.A., Li H., He Y., Fu J.Z. (2015). Quantitative analysis of surface profile in fused deposition modelling. Addit. Manuf..

[21] Buj-Corral I., Petit-Rojo O., Bagheri A., Minguella-Canela J. (2017). Modelling of porosity of 3D printed ceramic prostheses with grid structure. Procedia Manuf..

[22] Wang P., Zou B., Ding S. (2019). Modeling of surface roughness based on heat transfer considering diffusion among deposition filaments for FDM 3D printing heat-resistant resin. Appl. Therm. Eng..

[23] Bottini L., Boschetto A. (2019). Interference fit of material extrusion parts. Addit. Manuf..

[24] Popescu D., Zapciu A., Amza C., Baciu F., Marinescu R. (2018). FDM process parameters influence over the mechanical properties of polymer specimens: A review. Polym. Test..

[25] Domingo-Espin M., Puigoriol-Forcada J.M., Garcia-Granada A.A., Llumà J., Borros S., Reyes G. (2015). Mechanical property characterization and simulation of fused deposition modeling Polycarbonate parts. Mater. Des..

[26] Knoop F., Schoeppner V. Mechanical and Thermal Properties of Fdm Parts Manufactured with Polyamide 12. Proceedings of the Solid Freeform Fabrication Symposium.

[27] Uddin M.S., Sidek M.F.R., Faizal M.A., Ghomashchi R., Pramanik A. (2017). Evaluating Mechanical Properties and Failure Mechanisms of Fused Deposition Modeling Acrylonitrile Butadiene Styrene Parts. J. Manuf. Sci. Eng..

[28] Chacón J.M., Caminero M.A., García-Plaza E., Núñez P.J. (2017). Additive manufacturing of PLA structures using fused deposition modelling: Effect of process parameters on mechanical properties and their optimal selection. Mater. Des..

[29] Sood A.K., Ohdar R.K., Mahapatra S.S. (2012). Experimental investigation and empirical modelling of FDM process for compressive strength improvement. J. Adv. Res..

[30] McLouth T.D., Severino J.V., Adams P.M., Patel D.N., Zaldivar R.J. (2017). The impact of print orientation and raster pattern on fracture toughness in additively manufactured ABS. Addit. Manuf..

[31] Chaudhari M., Jogi B.F., Pawade R.S. Comparative Study of Part Characteristics Built Using Additive Manufacturing (FDM). Proceedings of the Procedia Manufacturing.

[32] Thrimurthulu K., Pandey P.M., Reddy N.V. (2004). Optimum part deposition orientation in fused deposition modeling. Int. J. Mach. Tools Manuf..

[33] Wang C.C., Lin T.W., Hu S.S. (2007). Optimizing the rapid prototyping process by integrating the Taguchi method with the Gray relational analysis. Rapid Prototyp. J..

[34] Ibrahim D., Ding S., Sun S. Roughness Prediction For FDM Produced Surfaces. Proceedings of the International Conference Recent Treads in Engineering & Technology (ICRET’2014).

[35] Li Y., Linke B.S., Voet H., Falk B., Schmitt R., Lam M. (2017). Cost, sustainability and surface roughness quality—A comprehensive analysis of products made with personal 3D printers. CIRP J. Manuf. Sci. Technol..

